# Effects of disturbances and environmental changes on an aridland riparian generalist

**DOI:** 10.7717/peerj.15563

**Published:** 2023-06-19

**Authors:** Brian R. Blais, Samantha L. Johnson, John L. Koprowski

**Affiliations:** 1 School of Natural Resources and the Environment, University of Arizona, Tucson, Arizona, United States; 2 College of Veterinary Medicine, University of Illinois at Urbana-Champaign, Champaign, Illinois, United States; 3 Haub School of Environment and Natural Resources, University of Wyoming, Laramie, Wyoming, United States

**Keywords:** Climate change, Conservation, Microhabitat, Noninvasive, Reptiles, Telemetry, Thamnophis, Desert, Gartersnake, Drought

## Abstract

Anthropogenic climate change and ecosystem disturbances can detrimentally affect habitats and species. Areas with concentrated biodiversity, such as aridland riparian zones, often yield the greatest number of vulnerable species. A better understanding of ecological and environmental relationships can guide more effective conservation strategies. We used both visual transects and external (tape) radio telemetry to study the behavioral and spatial ecology of black-necked gartersnakes (*Thamnophis cyrtopsis*; *n* = 81)—a dietary generalist yet aquatic habitat specialist—in a heterogenous aridland riparian zone of lower Sabino Canyon, Tucson, Arizona, between 2018 and 2021. Our objectives were to (1) understand how extrinsic conditions influence population ecology dynamics, including immediately prior to and after major disturbances and environmental extremes; (2) analyze behavioral activity and microhabitat usage in relation to environmental factors; and (3) assess the efficacy of a less-invasive telemetry strategy. Between late spring 2020 and early summer 2021, ecosystem disturbances included near-record heat and drought, wildfire, and low overwinter precipitation. Many aquatic habitats either completely dried or were spatially disjunct; gartersnake prey species were noticeably sparse. Extreme drought rapidly shifted to excessive flooding during the 2021 monsoon that brought above-average streamflow magnitude and duration. Between 2019 and 2021, we observed a dramatic decline in *T. cyrtopsis*; odds of detection reduced by 92.8% (CI [56.0–99.1%]). Strong spatiotemporal links relative to the extent and timing of available surface water appear important. Prior to the onset of monsoonal stream recharge in early summer, shallow and drying aquatic habitats are used as parturition sites and foraging grounds; all age classes took advantage to corral fishes trapped in isolated and shrinking pools. Ambient conditions had varying effects on gartersnake behaviors. Variation in microhabitat assemblages occurred with distance from water, activity level, and developmental age class. Interestingly, associations remained consistent across seasons and years, which suggests a reliance on heterogenous habitat structure. Sampling techniques complemented each other, however, bioclimatic parameters rendered limitations and should be considered in methodological decisions. Overall, disadvantageous responses to major disturbances and climatic extremes by a presumably adaptable generalist like *T. cyrtopsis* are concerning. Insights from long-term monitoring of responses by common yet environmentally sensitive species such as *T. cyrtopsis* may serve to more broadly highlight demographic challenges that other taxa with similar semi-aquatic life histories may face in changing systems. Such information could inform more effective conservation management strategies in warming and drying ecosystems.

## Introduction

Climate change-induced increases in temperature and aridity are predicted to continue while stressing water availability and demand (Pörtner et al., 2022), especially in aridland ecosystems (Archer & Predick, 2008; Roby, Scott & Moore, 2020). Anthropogenically-driven drought, wildfires, and stream disturbances, for example, have negatively impacted riparian habitats and their biodiversity in semi-arid regions of the southwestern United States (Desilets et al., 2007; Bogan, Boersma & Lytle, 2013; Tecle & Neary, 2015). Monitoring species responses to disturbances and assessing vulnerabilities to changing systems is essential for mitigating extinction risks and informing effective conservation management of natural resources (Dirzo et al., 2014; Pacifici et al., 2015).

Understanding the drivers of ecological and environmental relationships with natural and life history traits is also important for informing effective wildlife conservation strategies (Seddon, 2010; Robert et al., 2015). For example, abiotic conditions (*e.g*., temperature) and landscape features, such as distance to water and habitat patch characteristics, can influence distributional patterns and shape populations (Saupe et al., 2012; Bradie & Leung, 2017). Spatial ecology insights and understanding how species use landscapes is necessary to estimate movement and dispersal patterns (Peterson, 2006; DeAngelis & Yurek, 2017). Collectively, ecological information that spans multiple disciplines (*e.g*., behavioral and spatial ecology) can reveal how organisms respond to disturbances and inform effective conservation management strategies (Lytle & Poff, 2004; Merrick & Koprowski, 2017; Ingram et al., 2021).

Herpetofauna (*i.e*., amphibians and reptiles) are particularly threatened worldwide (Böhm et al., 2013; Roll et al., 2017; Cox et al., 2022). Because snakes in particular can facilitate substantial energy transfer between terrestrial and aquatic habitats, they make efficient models for assessing and upholding ecosystem integrity (Willson & Winne, 2016). Gartersnakes (*Thamnophis* spp.) are extensively studied and ideal reptiles to address broader ecological questions (Rossman, Ford & Seigel, 1996; Gignac & Gregory, 2005; Hallas, Parchman & Feldman, 2022). Several gartersnake species are riparian specialists and sensitive to habitat alterations or shifts in trophic levels, thus are useful to investigate how environmental changes may impact sensitive wildlife (Huey et al., 1989; Gregory, 2001; González-Fernández et al., 2018)—especially in warming and drying semi-arid riparian systems (Griffis-Kyle et al., 2018; Bateman & Riddle, 2020).

The black-necked gartersnake (*Thamnophis cyrtopsis*) is relatively common and widely distributed from the southwestern United States to Guatemala (Rossman, Ford & Seigel, 1996; Ernst & Ernst, 2003). Considered a dietary generalist but aquatic habitat specialist (Jones, 1990), *T. cyrtopsis* are often linked to lotic and rocky riparian networks at varied elevations in montane desert areas of its northern range (Rossman, Ford & Seigel, 1996; Jones & Hensley, 2020). Anurans and fish are common in its diet and adaptive foraging strategies appear linked to seasonal environmental changes (*e.g*., from dry spring to wet summer monsoon) and natural history, such as capitalizing on certain prey bases when they are most vulnerable (Fleharty, 1967; Jones, 1990). Despite its common status, population declines of *T. cyrtopsis* have been associated with disturbances and rapid environmental changes (Rorabaugh & Lemos-Espinal, 2016; Jones & Hensley, 2020). Environmental vulnerabilities suggest that *T. cyrtopsis* represents a model system for evaluating responses to climate change in aridland riparian systems, including rapid disturbances and environmental extremes.

In this study, we used both radio telemetry and visual encounter survey techniques to monitor the behavioral and spatial ecology of *T. cyrtopsis* in a semi-arid riparian system. Our objectives were to (1) obtain preliminary insights into *T. cyrtopsis* population ecology dynamics; (2) examine the spatial and behavioral ecology of *T. cyrtopsis*—specifically *via* age class, activity, and microhabitat use associated with seasonal and environmental conditions; and (3) assess the efficacy of a less-invasive monitoring strategy (“tape telemetry”; Wylie et al., 2011). We hypothesized that we would detect more surface-active *T. cyrtopsis* during the summer monsoon season relative to streamflow recharge; that there would be a strong relationship between behavioral activity and surface water; and that microhabitat associations would vary seasonally (*i.e*., composition types used in the rainy summer season *vs*. the dry spring and fall seasons) and by detection method. During our study, the site experienced substantial disturbances, such as effects from intense wildfire, extreme heat and drought, and above average flood/flow regime. This presented an opportunity to (4) investigate immediate responses in a recently disturbed riparian ecosystem. A noticeable decline in observations would likely reflect similar trends and concerns observed elsewhere, such as population declines attributed to drought and reduction in available resources (*e.g*., prey, aquatic habitat; Jones & Hensley, 2020). Although considered common, *T. cyrtopsis* movement ecology is relatively unknown (Ernst & Ernst, 2003; Jones & Hensley, 2020), and this study advances knowledge of its natural history and spatial ecology. The ecological and life history insights from this common species may serve to highlight challenges that other taxa with similar semi-aquatic life histories may face in warming and drying ecosystems.

## Materials and Methods

### Study site

Sabino Canyon Recreation Area (SCRA) is located at the southern base of the Santa Catalina Mountains in Tucson, Arizona, USA (Lazaroff, 1993). The region experiences a semi-arid Sonoran Desert climate with bimodal seasonal precipitation (Desilets et al., 2007; Webb et al., 2008). Although Sabino Creek is considered a perennial headwater stream in its upper reaches (Bogan et al., 2020), lower Sabino Creek is seasonally intermittent—wetted during the winter and early spring months before drying up and leaving disjunct pools during the hot, dry period prior to summer monsoonal recharge (Lazaroff, 1993; Lazaroff, Rosen & Lowe, 2006). Sonoran desertscrub (saguaro-palo verde communities) surrounds the heterogeneously complex riparian woodland-mesquite bosque biotic community adjacent lower Sabino Creek (Lazaroff, 1993). Streambed and floodplain substrates range from fine sands and cobbles to boulders, and in some parts of the canyon, exposed bedrock. Riparian vegetation spans low herbaceous plants, shrubs, and tall deciduous overstory trees (*e.g*., *Fraxinus*, *Platanus*, *Populus*; Lazaroff, 1993); debris and sediment deposits from high flow events can influence microhabitat composition (Desilets et al., 2007). The SCRA has a rich herpetofauna with more than 50 species, and the most frequently observed snake is *T. cyrtopsis* (Lazaroff, Rosen & Lowe, 2006).

The study area has also experienced recent environmental disturbances and anthropogenic effects. Contemporary ground water demands have negatively impacted Tucson-area watersheds (Carlson et al., 2011; Bogan et al., 2020). Effects from the Bighorn Wildfire (Wilder et al., 2021) resulted in ash slurry deposits throughout lower Sabino Creek and within many pool microhabitats (B Blais, 2020, personal observations; see Fig. S1). Near-record heat and drought occurred in 2020 (McMahan et al., 2021) and continued into mid-summer 2021 that left few disjunct pools with surface water during that time (B Blais, 2021, personal observations). Beginning in late July 2021, monsoon precipitation brought unusually persistent and relatively high discharge magnitude (mean daily flow (June–September 2021) = 1.24 m^3^/s, prior 10-year mean = 0.15 m^3^/s; https://waterdata.usgs.gov/nwis/). Safety issues due to the Bighorn fire and the onset of COVID-19 restricted our access to SCRA between April–September 2020, and flash flooding and high flows also limited access to certain study reaches during the 2021 monsoon.

### Experimental design

We used *a priori* knowledge of *T. cyrtopsis* (Jones, 1990; Rossman, Ford & Seigel, 1996; Jones & Hensley, 2020) to guide visual encounter surveys (VES; Doan, 2016) in riparian habitat adjacent (±50 m) lower Sabino Creek in the SCRA between 2018 and 2021. Because *T. cyrtopsis* is primarily diurnal (Lazaroff, Rosen & Lowe, 2006; Jones & Hensley, 2020), we began most surveys in the morning hours (mean = 09:00) but occasionally surveyed in early evenings during the monsoon season (mean night start ca. 17:30). At the start and end of each survey, we used an anemometer (Kestrel 5000; Nielsen-Kellerman, Boothwyn, PA, USA) to record mean ambient conditions for air temperature (TA; ±0.1 °C), barometric pressure (BP; ±0.1 millibars), relative humidity (RH; ±0.1%), and wind speed (±0.1 m/s). We nominally categorized precipitation (*e.g*., none, rain ≤24 h) and we obtained maximum stream flow discharge (m^3^/s) during survey windows from the USGS Sabino Creek gage (https://waterdata.usgs.gov/monitoring-location/09484000/).

We hand-captured *T. cyrtopsis* and recorded body size (snout-vent length [SVL; ±0.1 cm], tail length [TL; ±0.1 cm], and body mass [±0.1 g]) and sex when applicable. We classified developmental age classes of *T. cyrtopsis* as follows: <200 mm SVL = neonates; females >411 mm and males >337 mm SVL = mature adults; and intermediate SVL sizes = immature/subadults (Jones & Hensley, 2020; Dataset S1). For mark-recapture purposes, we used a low-temperature cauterizer (DEL0; Bovie Medical Corp., Clearwater, FL, USA) to code-brand individuals (Winne et al., 2006) and injected a PIT-tag (HPT9; Biomark Inc., Boise, ID, USA) subcutaneously (Bronikowski & Arnold, 1999; Moore & Gillingham, 2006). We omitted sexing or marking neonates due to their size. We recorded ambient environmental conditions for each snake encountered, whether captured or not. Fieldwork was conducted under the University of Arizona’s Institutional Animal Care and Use Committee (IACUC protocol #16–169) and scientific research permits from Arizona Game & Fish Department and USDA Forest Service (SUP1841).

### Behavior and microhabitat usage

We recorded each individual’s displayed behaviors (*ad libitum*) between first detection until observer approach or capture (ca. ≤2 min; Pelegrin et al., 2017). Specifically, we binarily scored behaviors of individuals prior to any reactions to approaching observers. We based behaviors (*e.g*., inactivity, alertness, moving, water use, and foraging) upon known attributes of conspecifics (Rossman, Ford & Seigel, 1996; Lillywhite, 2014; Blais et al., 2022) and *T. cyrtopsis* specifically (Fleharty, 1967; Jones, 1990; Jones & Hensley, 2020; behaviors are described in Dataset S1). Individuals could display >1 behavior. We considered a snake to be surface-active if it was visible above-ground or in water, *i.e*., not in a burrow or hidden within terrestrial refuges (Hyslop, Cooper & Meyers, 2009).

For microhabitat use, we assessed 1 m diameter circular plots at detection sites (Sprague & Bateman, 2018) to estimate percent ground composition for the following variables: open (*i.e*., barren), rock (*e.g*., sand through boulder/bedrock), woody debris (*e.g*., detritus, plant litter), grasses/forbs, shrubs, trees (including exposed/eroded root systems), and water. We averaged four radial readings from a 17-point densiometer (Strickler, 1959) to estimate overhead canopy cover percentage. We also categorized vegetation height classes above plots as well as distance to nearest surface water (Dataset S1). When plots were in or near (≤1 m) a waterbody, we binarily recorded the corresponding streamflow classification types (*e.g*., plunge pool; backwater and isolated pools; runs/glides; riffles; water’s edge; stream bank; and when applicable, dry bed). We noted the presence of potential vertebrate prey (*e.g*., anurans, fishes) at aquatic microhabitats but did not quantify prey characteristics.

### Tracking

Radio telemetry effectively provides resolution into the spatial ecology and life history traits of snakes (Stanford, King & Wynn, 2010; Kingsbury & Robinson, 2016). For a subset of adequately sized *T. cyrtopsis*—estimated by girth and body mass (≥44 g, *i.e*., 5% transmitter mass ratio; Kingsbury & Robinson, 2016)—we employed an external “tape” telemetry method used with other *Thamnophis* (Wylie et al., 2011; Sprague & Bateman, 2018). This less invasive method provides short-term monitoring until snakes shed. We cut a thin band of duct tape (Scotch® 3M; All-Weather, Sacramento, CA, USA) to attach a 2.2 g VHF transmitter (IMP-CHP-7P; Telonics, Inc., Mesa, AZ, USA) ventrally on the posterior third of a snake but anterior of the cloaca. This positioning allowed the transmitter to press unto soft muscle and not protrude over bone or drag behind the tail (Wylie et al., 2011). We processed and released all gartersnakes *in situ* at capture sites.

We used a directional VHF antenna and receiver (RA-23K, R-1000; Telonics, Inc., Mesa, AZ, USA) to geolocate snakes *via* homing with attempted accuracy ≤10 m, when applicable, and noted if individuals were active on the surface or hidden within refuges (Smith, Spotila & Bien, 2015). We relocated individuals on average every 4.4 (SD ±2.9) days between 07:30–20:00. We recorded all data similarly as VES captures except for microhabitat composition if the individual moved less than three meters from its prior location (Sprague & Bateman, 2018). We terminated tracking when we found a shed/dropped transmitter, deceased snake, or when the locality point did not change for ≥21 days during the active season.

### Analyses

We first used Principal Component Analysis (PCA) in the R software package FactoMineR (Lê, Josse & Husson, 2008) to assess the following survey-level quantitative variables: survey duration (minutes), max stream flow, and the minimum and maximum values for ambient BP, RH, TA, and wind. Because *T. cyrtopsis* water usage may be influenced by flow dynamics relative to the monsoon season (*e.g*., drying pools *vs*. contiguous reaches; Jones & Hensley, 2020), we partitioned data seasonally (pre-monsoon = January–June; monsoon = July–September; and post-monsoon = October–December) and used this factor and *T. cyrtopsis* survey presence (binary) as grouping variables. This process served to optimize covariate selection to the most contributing and representative variables from the PCA while reducing multicollinearity (*e.g*., min and max values of a variable were highly correlated, Pearson’s *r* > 0.8). From the PCA results, we used survey minimums for RH, TA, and wind and maximum BP for downstream analyses.

Next, we performed multiple logistic regression (*i.e*., generalized linear models, GLM) for survey-level presence with iterations of predictor-fitted candidate models and a null intercept-only model (Venables & Ripley, 2013). The full model included the following predictors: BP, RH, TA, wind, stream flow, survey duration, season, and year. We used backwards elimination to remove uninformative parameters (Leroux, 2019) and selected models based on corrected Akaike’s information criterion (AICc) values in the AICcmodavg package (Mazerolle, 2020). We inspected models for variable inflation (VIF) and overdispersion; we converted top model coefficients to odds ratios with 95% confidence intervals. Because surveys in 2018 were exploratory and surveys in 2020 were limited due to site closures, we only modeled data for 2019 and 2021 surveys. Similarly, we repeated the GLM process but with a Poisson distribution to understand potential drivers of *T. cyrtopsis* abundance (here, the quantity of individuals observed per survey). We report McFadden’s pseudo-R squared scores (*i.e*., proportional increase in model fit with predictors added) and maximum log likelihoods for all models. We visualized predicted marginal effects from coefficients in the sjPlot package (Lüdecke, 2021).

We used logistic GLMs to understand influences of surface activity for the following behaviors, separately: alertness, foraging, inactivity, movement (*i.e*., locomotion), and water usage (*i.e*., swimming, floating). Full models included age class, TA, RH, BP, wind, rain, distance to water (except for water usage), and season. Because we observed few immature gartersnakes, we combined immatures and neonates as “juveniles” for analyses hereafter. We used Fisher’s Exact Tests for categorical associations. We used hierarchical clustering *via* Gower’s distance metric in R package cluster (Maechler et al., 2021) to calculate dissimilarity distances among stream reach types.

We considered use of surface-level microhabitats by *T. cyrtopsis* as a function of selected ecological patterns (Hyslop, Cooper & Meyers, 2009); it was not feasible to assess underground microhabitats or those within complex refuges. We performed factor analysis of mixed data (FAMD) in FactoMineR to first visualize normalized associations among quantitative and qualitative variables of microhabitats. Qualitative variables included distance to water, season, tracking method, vegetation height (cover) class, and year; we designated age class and surface-activity as supplementary variables. We omitted microhabitat composition variables with excessive zeroes (>80%). Although principal components (PCs) with eigenvalues >1 are considered important (Hyslop, Cooper & Meyers, 2009; Sprague & Bateman, 2018), we retained all PCs to not omit important correlations with dependent variables in downstream analyses (Lee, Park & Lee, 2015). We investigated each variable’s PC contributions and visualized them in factorial biplots. From the FAMD results, we used logistic regressions to assess the most contributing and representative factors against the response variables, surface-activity and age class, separately. We also used this process to examine if detection method (*i.e*., telemetry *vs*. VES) would parse out differences in microhabitat use.

We used the packages adehabitatLT and adehabitatHR (Calenge, 2006) to summarize descriptive spatial statistics of distances moved per day during tracking windows as well as to estimate intraseasonal home ranges *via* minimum convex polygons for individuals with at least five relocations (Row & Blouin-Demers, 2006). For individuals with undetermined outcomes (*e.g*., unrecoverable transmitter; inaccessible final location), we omitted localities beyond 10 days of no movement. This span accounts for the approximate duration of ecdysis (*i.e*., shedding) within refuges (Lillywhite, 2014); shedding is the most likely outcome for tracking cessation with tape telemetry (Wylie et al., 2011). We performed all analyses in R v.4.1.1 (R Core Team, 2021) and report means ±1 standard deviation and significance at α = 0.05 for all tests unless otherwise specified. We used the R packages ggmap (Kahle & Wickham, 2013) and sp (Bivand, Pebesma & Gomez-Rubio, 2013) to generate the sampling map.

## Results

### General results and survey-level trends

**Figure 1:**
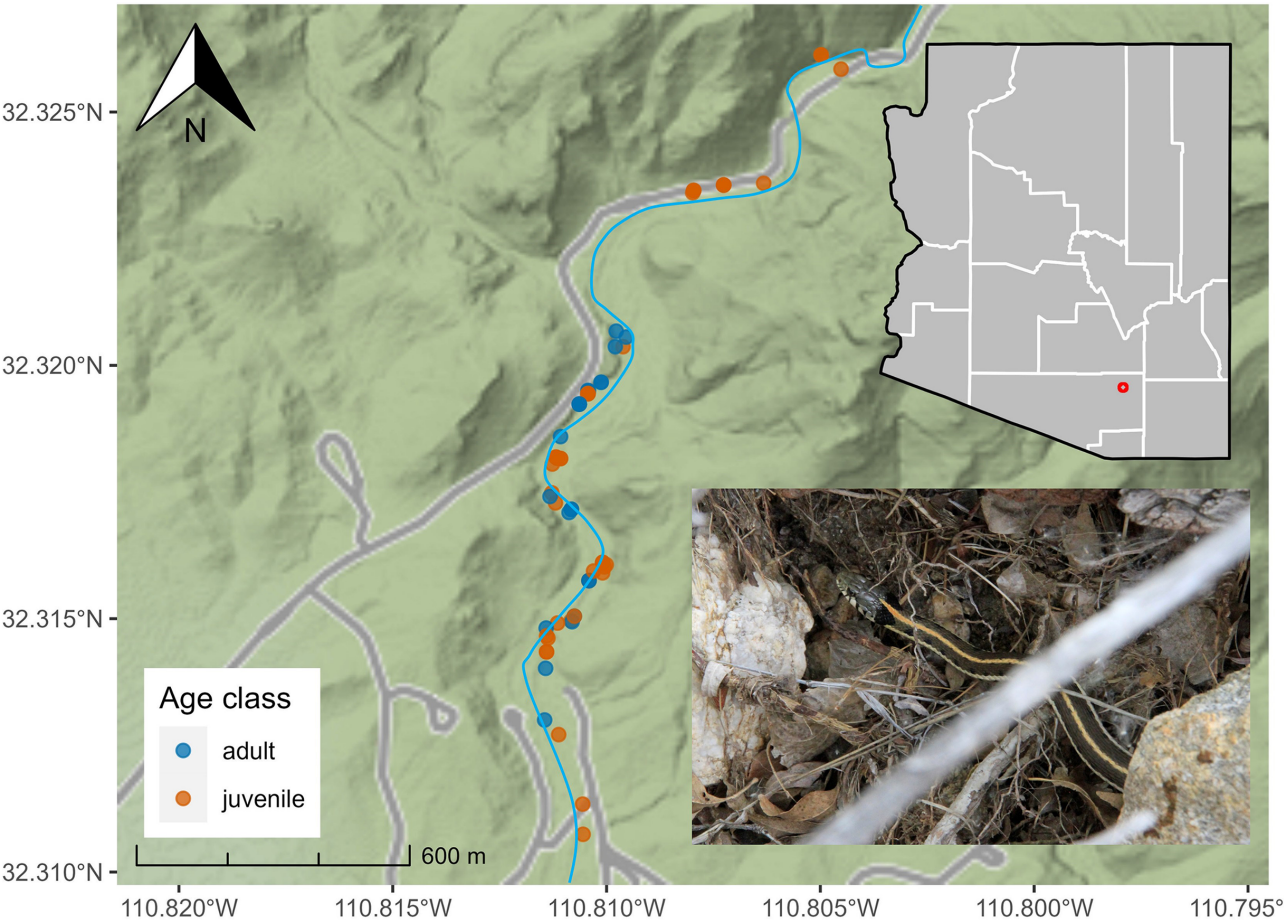
Localities of black-necked gartersnakes (*Thamnophis cyrtopsis*; inset bottom right) in Sabino Canyon Recreation Area, Tucson, Arizona, 2018–2021. Points are colored by developmental age class. Grey lines represent paved, private service roads in the SCRA. Sabino Creek (blue) is approximated as the channel and surface volume shift seasonally and annually. Map data © Stamen Maps.

Between July 2018 and December 2021, we conducted 71 surveys in lower SCRA (seasons: pre-monsoon = 14, monsoon = 35, post-monsoon = 22) and collected data on 81 *T. cyrtopsis* (*n* = 29 adults, *n* = 52 immature/neonates). We detected *T. cyrtopsis* age classes equivalently throughout the sampling area (Fig. 1). We amassed body size data from 25 individuals. Adult SVL averaged 481 mm (SD ±38) and body mass averaged 49.0 g (SD ±11.9; Table 1). Adult females trended longer but not heavier than males, and relative body condition (*i.e*., log(mass)/log(SVL); Stevenson & Woods, 2006) were not different between sexes (*t* = −0.34, df = 12, *p* = 0.743). The ratio of SVL to tail length was 24.3% (SD ±1.7%), equivalent among age classes (*t* = −1.13, df = 6, *p* = 0.301).

**Table 1:** Body sizes of black-necked gartersnakes (*Thamnophis cyrtopsis*) in Sabino Canyon Recreation Area, Tucson, Arizona, 2018–2021.

Age class, sex	SVL (mm)	Mass (g)	BCI
Adult, female (*n* = 6)	498 ± 51	49.2 ± 13.5	0.623 ± 0.039
Adult, male (*n* = 8)	468 ± 24	49.4 ± 12.4	0.630 ± 0.038
Adults* (*n* = 15)	481 ± 38	49.0 ± 11.9	0.626 ± 0.036
Immature, unsexed (*n* = 3)	345 ± 64	15.3 ± 5.4	0.456 ± 0.074
Neonates, unsexed (*n* = 7)	198 ± 20	3.7 ± 1.1	0.252 ± 0.053

**Notes:**

* Includes both sexes and one unsexed individual.

SVL, snout-vent length in millimeters; BCI, Relative body condition index is log of mass divided by log of SVL; see Stevenson & Woods (2006). Units are mean ±1 standard deviation.

We observed fish foraging behaviors at mostly shallow but variably sized pools. Several *T. cyrtopsis* in the SCRA demonstrated a fascinating foraging strategy near the edge of very shallow and receding pools (ca. ≤5 cm depth) in which they used their bodies like seines to corral and isolate small or young fish (*e.g*., Gila chub, *Gila robusta* complex; Gila topminnow, *Poeciliopsis occidentalis*) between themselves and the shore before striking at individual fish. Though we did not quantify strike success, this strategy appeared effective at fish capture; see *figshare* media in Blais (2023). We observed this behavior in adults and neonates during periods prior to stream recharge. Foraging at shallow depths or near the surface of water is known for this generalist (Fleharty, 1967; Jones & Hensley, 2020), but the seine-like foraging strategy differs from what has been described (*e.g*., ambush from floating algal mats, Jones, 1990; Jones & Hensley, 2020). Although we did not observe foraging for frogs, some individuals regurgitated anurans during processing, including a juvenile metamorphosed American bullfrog (*Lithobates catesbeianus* [nonnative], SUL ≤5 cm) and several ca. 10–20 mm early-stage tadpoles (likely canyon treefrog, *Hyla arenicolor*—the most frequent anuran encountered during the study). We also observed a neonate *T. cyrtopsis* being predated by a regal ring-necked snake (Blais & Binford-Walsh, 2020). Defensively, most gartersnakes attempted to flee from capture, and did not hesitate to musk once in hand.

**Figure 2:**
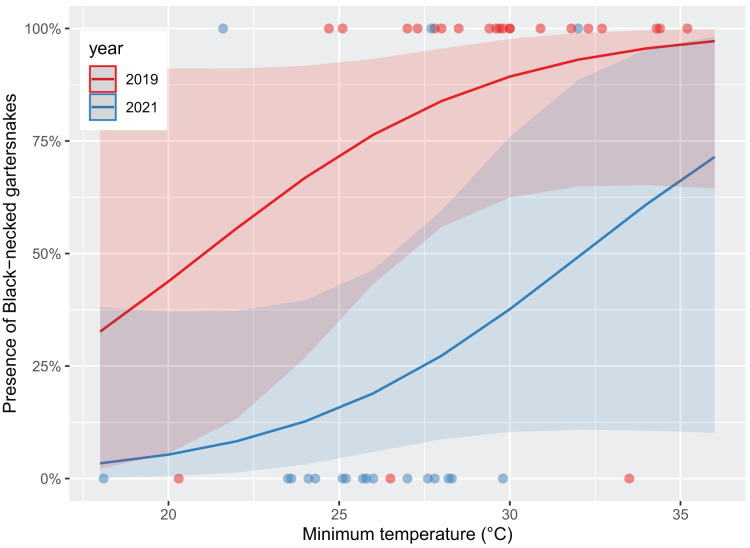
Likelihood of black-necked gartersnakes (*Thamnophis cyrtopsis*) presence during surveys in Sabino Canyon Recreation Area, Tucson, Arizona, 2019 & 2021. Logistic regression models for predicted likelihood of presence, proxied as at least one individual observed per visual encounter survey. Colors depict sampling year. Presence was influenced by sampling year with trending influence from survey minimum ambient temperature (±0.1 °C). Data from 2020 was omitted due to restrictions from COVID-19 and the Bighorn Wildfire.

**Figure 3:**
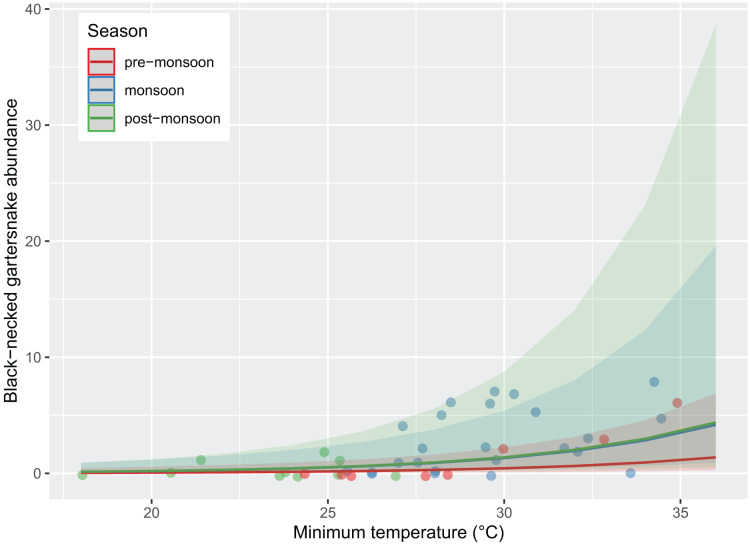
Abundance of black-necked gartersnakes (*Thamnophis cyrtopsis*) observed during surveys in Sabino Canyon Recreation Area, Tucson, Arizona, 2019 & 2021. Predicted likelihood of abundance, proxied as the total number of individuals observed per visual encounter survey. Abundance was driven by season (colors) and survey minimum ambient temperature (±0.1 °C). Data from 2020 was omitted due to restrictions from COVID-19 and the Bighorn Wildfire.

Survey presence of *T. cyrtopsis* was best explained by a model with the covariates, year, TA, and survey duration (McFadden’s *R*^*2*^ = 0.505; Table 2). The odds of detection on a survey reduced 92.8% (95% CI [56.0–99.1%]) in 2021 compared to 2019 after accounting for other covariates (Fig. 2). Survey abundance of *T. cyrtopsis* in 2019 (mean ± standard error [SE] = 3.48 ± 0.53; per person visit = 1.96 ± 0.43) was greater than in 2021 (mean ± SE = 0.43 ± 0.24; per person visit = 0.20 ± 0.12). There were three models with AICc <2 that best explained abundance per survey (Table 2). All models contained TA, duration, season, and year but varied by BP and streamflow (*R*^*2*^ = 0.478–0.506). A shift from pre-monsoon to monsoon increased the odds of observing more individuals by 3.04 times (CI [1.49–6.79]; Fig. 3). Also, for each unit change in TA, the odds of greater abundance increased by 21.3% (CI [8.9–36.2%]). Though identified in the top models, the impacts of BP, survey duration, or flow on abundance were negligible or with uncertainty (*i.e*., <1% shift in odds, confidence intervals crossed zero).

**Table 2:** Extrinsic influences on per survey detection and abundance of black-necked gartersnakes (*Thamnophis cyrtopsis*) in Sabino Canyon Recreation Area, Tucson, Arizona, 2018–2021. Selection of generalized linear models was performed using corrected Akaike Information Criterion (∆AICc <2). Table is split by (A) logistic regression for survey presence, *i.e*., detection; and (B) Poisson regression for abundance per survey. *Model* represents the model covariates; *k* is the number of model parameters; *AICc* is corrected AIC score; ∆*AICc* is the change in AICc scores; *loglik* is the model maximum log likelihood; and *R^2^* is McFadden’s pseudo r-squared value.

	Model	k	AICc	∆AICc	loglik	R^2^
A) Presence	~ TA + dur + year	4	37.73	0.00	−14.32	0.51
	~ TA + flow + dur + season + year	7	40.61	2.88	−11.66	0.60
	~ TA + dur + season + year	6	41.50	3.77	−13.15	0.53
	~ RH + TA + flow + dur + season + year	8	43.29	5.57	−11.47	0.60
	~ RH + TA + w + flow + dur + season + year	9	45.22	7.49	−10.80	0.63
	~ dur + year	3	45.34	7.61	−19.41	0.44
	~ RH + BP + TA + W + flow + dur + season + year	10	48.54	10.81	−10.72	0.63
	~ 1	1	71.08	33.35	−34.50	—
B) Abundance	~ TA + dur + season + year	6	118.64	0.00	−52.12	0.48
	~ BP + TA + flow + dur + season + year	8	118.90	0.25	−49.27	0.51
	~ BP + TA + dur + season + year	7	119.64	1.00	−51.17	0.49
	~ RH + BP +TA + flow + dur + season + year	9	122.16	3.51	−49.27	0.51
	~ TA + dur + year	4	122.73	4.08	−56.82	0.43
	~ RH + BP + TA + W + flow + dur + season + year	10	125.61	6.97	49.26	0.51
	~ dur + year	3	177.98	59.34	−85.73	0.32
	~ 1	1	252.83	134.18	−125.37	—

**Note:**

*Model parameter abbreviations*: BP, ambient barometric pressure (millibars); dur, survey duration (minutes); RH, ambient relative humidity (%); flow, maximum stream flow (m^3^/s); TA, ambient temperature (°C); season, seasonality (pre-monsoon, monsoon, post-monsoon); W, ambient wind speed (m/s). Covariates are described in Dataset S1.

### Activity and behavior

Most *T. cyrtopsis* observations occurred during the monsoonal months, July–September (71.8%). We first observed neonates in late June. Most detections occurred mid-morning to early-afternoon, but we did observe individuals (all age classes) active in early evenings (*e.g*., 18:00–20:00) during the monsoon season. This aligns with seasonally cathemeral activity patterns known among other *T. cyrtopsis* populations (Jones & Hensley, 2020). Gartersnakes were surface-active in intermediate ambient temperatures (median = 32.2 °C; Fig. S2A) and with increasing relative humidity (median = 31.3%; Fig. S2B). Ambient environmental conditions at sightings are summarized in Table S1.

**Figure 4:**
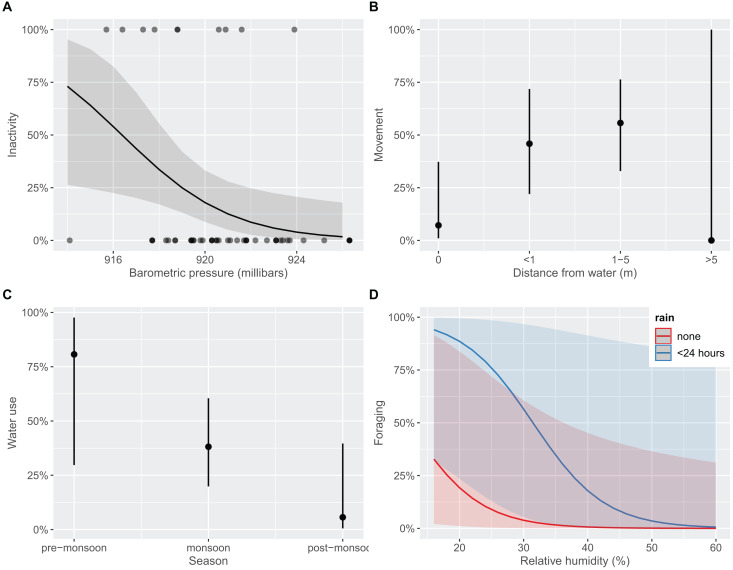
Predicted marginal effects of extrinsic drivers on specific behaviors of black-necked gartersnakes (*Thamnophis cyrtopsis*) in Sabino Canyon Recreation Area, Tucson, Arizona, 2018–2021. Predicted likelihood of outcomes from logistic regression on the presence of behaviors as influenced by extrinsic variables (y-axes). Behaviors include (A) inactivity (*e.g*., basking, refuge use), (B) movement (*i.e*., terrestrial locomotion), (C) water use (*e.g*., floating, swimming), and (D) foraging (*i.e*., evidence of eating). Behaviors are described in Dataset S1.

We found behavioral differences between age classes for inactivity (favoring adults; Fisher’s Exact Test *p* < 0.001), foraging (favoring juveniles; *p* = 0.021), and water use (favoring juveniles; *p* = 0.001; Table S2); movement or alert behaviors were observed equivalently between adults and juveniles (*p* > 0.05), respectively. The top model to explain inactivity included BP and TA (*R*^*2*^ = 0.162; Table S3). After accounting for effects of TA, each unit increase in BP decreased the odds of observing inactivity by 34.3% (CI [9.7–56.5%]; Fig. 4A). That is, *T. cyrtopsis* were more likely to be inactive as pressure dropped. Inactivity also trended positively with unit increases in ambient temperature (25%), but with much variability (CI [−1.5 to 67.0%]). Two competing models best explained gartersnake movement (AICc <2); both included distance to water but differed by BP (*R*^*2*^ = 0.232) or RH (*R*^*2*^ = 0.233). In either case, observing a gartersnake in motion (terrestrially) increased when close to water but not beyond 5 m from water (Fig. 4B). Observing alert behaviors were best explained by season, rain, RH, and wind (*R*^*2*^ = 0.525; Table S3). Observations of alert behaviors were less likely to occur during the wet monsoon season than the flanking dry seasons (Fisher’s Exact Test, *p* < 0.001). Rain, RH, or wind were uninformative towards alertness, *i.e*., odds ratios confidence intervals crossed zero. Seasonality and TA best explained water usage (*R*^*2*^ = 0.148) albeit a competing model that included age class performed equivalently (*i.e*., AICc <2, *R*^*2*^ = 0.161; Table S3). Observations of water use declined with each subsequent season (Fig. 4C). For instance, 80% of observations during the pre-monsoon period included some form of water use, dropping to 56.1% during the monsoon season, and just 9.1% during post-monsoon (Fisher’s Exact Test, *p* = 0.002). Finally, foraging revealed three competing models within 2 AICc units (Table S3). Common covariates were distance to water, season, and RH (*R*^*2*^ = 0.378) whereas models differed by age class, BP, rain, and TA (*R*^*2*^ = 0.497–0.582). We interpret these findings as foraging observations were more likely to occur by juveniles (Fisher’s exact test, *p* = 0.021), in water (*p* < 0.001), and when rain occurred within 24 h (*p* < 0.001), and least likely to occur during the post-monsoon season (*p* = 0.014). Also, with each unit increase in RH, the odds of foraging declined by 16.3% (CI [3.5–32.5%]; Fig. 4D).

### Microhabitat use

*Thamnophis cyrtopsis* used a variety of microhabitats. When inactive or not foraging, refuges often consisted of exposed tree root systems, debris piles from high-flow events, and rock piles; these trends are similar to other *T. cyrtopsis* populations in Arizona (Jones, 1990). We seldom recorded use in barren microhabitats or those with grasses/forbs or shrubs. Of applicable stream reach types used or adjacent plots, pool microhabitats comprised the predominant cluster (64.6% of all observations); these consisted of backwater or still (*e.g*., isolated) pools (Fig. S1). Seldom used were glides, riffles, or plunge pools (<10%), though the latter were uncommon in our survey area; we also less often observed gartersnakes at larger, deeper pools. When near water, most *T. cyrtopsis* used shallow aquatic habitats (depth mean ± SD = 9.0 ± 7.3 cm) or the water’s edge (36.9% of applicable observations). Only twice did we record individuals in water beyond one meter from the shore (max distance = 7.8 m), both occurrences were neonates. We note that some data were recorded during complete flow cessation and many stream reach types, save for a few isolated pools, were not present.

**Figure 5:**
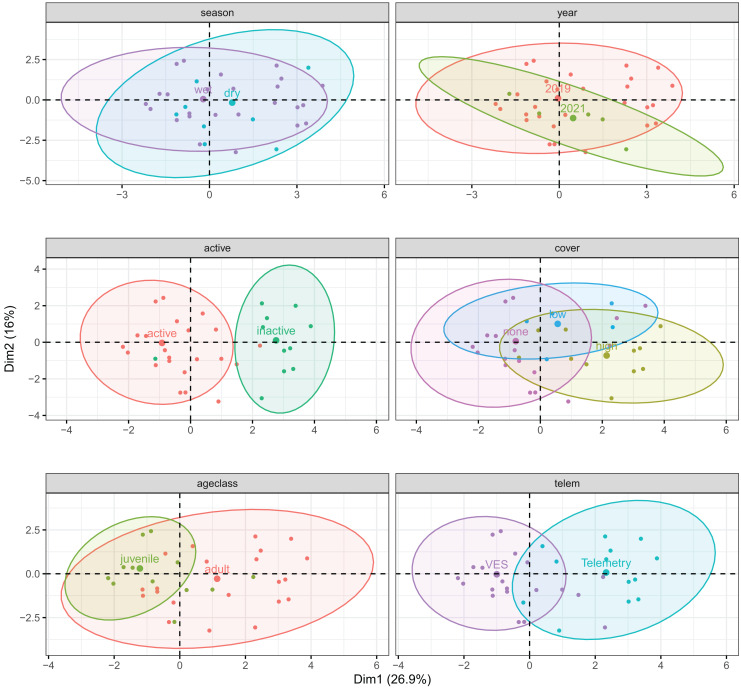
Correlation biplots of microhabitat composition used by black-necked gartersnakes (*Thamnophis cyrtopsis*) in Sabino Canyon Recreation Area, Tucson, Arizona, 2018–2021. Graphs are derived from factorial analysis of mixed data (FAMD) and are subdivided by qualitative factors (colors, labels); points depict spread of individually used microhabitats assuming multivariate t-distributions. Ellipses are centered around qualitative group means (larger points). The first three principal components explained 57.2% of microhabitat variance.

The first three principal components explained 57.2% of microhabitat variance (Table S4). Percent water (in 1 m plots) and distance to water contributed most to PC1 (26.9% variance); distance to water and percentages of rock and trees/roots contributed most to PC2 (16.0%); and distance to water, cover, and shade contributed most to PC3 (14.3%). Overall, composition of microhabitats used by *T. cyrtopsis* were largely driven by percentages of rocky substrate, shade, and water but not canopy density. Shaded microhabitats tended to be less rocky and less adjacent to surface water; such areas were correlated to trees and woody debris. Among qualitative variables, there were discernable sublevel clusters for surface-activity, detection method, and to an extent, age class (Fig. 5). That is, microhabitat composition was at least partially varied among active *vs*. inactive gartersnakes; adults *vs*. juveniles; and *via* telemetry *vs*. those detected by VES. Interestingly, compositions of microhabitat characteristics were not dissimilar by year (*i.e*., 2019 *vs*. 2021) or season (dry *vs*. wet; Fig. 5).

The top model to describe differences in microhabitat composition by *T. cyrtopsis* activity (*vs*. inactivity, *i.e*., refuge use) included plot percentages of canopy cover, rock, shade, trees/roots, water, and woody debris (McFadden’s *R*^*2*^ = 0.647; Table S5). Observed gartersnakes were nearly always active when within one meter of water (97.5%) but there was uncertainty among other model covariates, *i.e*., odds ratios crossed zero. The top model to detect microhabitat differences by age classes included mean canopy as well as plot percentages of shade, rock, trees/roots, water, and woody debris (*R*^*2*^ = 0.348; Table S5). With each unit increase in percent canopy cover, the odds of a site being used by a juvenile declined 93.0% (CI [68.0–99.9]) than an adult after accounting for other covariates. In summary, we conservatively interpret used microhabitat composition by activity level and age class as follows: active snakes tended to be closer to water and in rockier areas, especially juveniles, whereas inactive sites were likely to be relatively more shaded and near woody debris, especially those used by adults. Rock and trees/roots usage were relatively indistinguishable between age classes and dense overhead canopy appeared to be generally avoided.

Three models best described differences in detection method. Among all three were percentages of water and shade and they differed by canopy cover and woody debris (*R*^*2*^ = 0.433–0.443; Table S5). Compared to telemetry, the use of VES increased the odds ratio for detecting gartersnakes in or within one meter from water by 177.2 times (CI [5.8–1.05^+4^]) and plots were 89.3% (CI [0.9–90.8]) less shaded. Accordingly, technique also differed in distance classes to water (Fisher’s Exact Test, *p* < 0.001) and for detecting inactive gartersnakes (*p* < 0.001). To summarize, telemetry was more efficient at finding inactive gartersnakes in shadier places (*e.g*., more vegetated microhabitats, refuges) whereas VES was effective along waterways.

### Seasonal movement and spatial ecology

We acquired telemetry data for six adult *T. cyrtopsis* (three females, three males); mean transmitter to body mass ratio was 3.9% (±0.8%). Site access restrictions and difficulty detecting adequately sized gartersnakes limited tracking to only 2019. Tracking duration varied (range: 5–54 days, mean ± SD = 20 ± 17.8; Table 3). Because sample size was limited, we report movement and relative intraseasonal home range inferences (Sacerdote-Velat et al., 2014) and omit partitioning by sex. Mean daily movement distances trended increasingly with tracking duration (*F* = 3.84, *p* = 0.122, *R*^*2*^ = 0.363; Fig. S3); most movements were minimal (*e.g*., <10 m). Mean home range was 0.28 ha (±SD 0.52 ha), though the limited span of data likely underestimates annual home range size. The furthest any tracked individual was detected from the primary stream channel was ca. 117 m. We had two non-telemetry recaptures during our study. An adult male was recaptured on 20 September 2019 after 71 days and was 11.0 g heavier than first capture; another adult male was recaptured on 7 November 2019 after 48 days and was 7.5 g lighter.

**Table 3:** Intraseasonal spatial ecology metrics for radio-tracked adult black-necked gartersnakes (*Thamnophis cyrtopsis*) in Sabino Canyon Recreation Area, Tucson, Arizona, 2019.

Individual (body size)	Sex	Date (duration)	Dist (max)	Dist (mean ± SD)	MCP (ha.)
THCY004 (551 mm SVL, 43.2 g)	F	3 July (7)	10.6	8.1 (3.5)	—
THCY005 (565 mm SVL, 71.0 g)	F	3 July (15)	5.0	1.0 (2.2)	<0.001
THCY008 (450 mm SVL, 57.7 g)	F	22 August (19)	12.4	4.8 (6.0)	0.014
THCY002 (475 mm SVL, 52.2 g)	M	28 June (20)	18.0	2.9 (6.7)	0.042
THCY003 (475 mm SVL, 54.2 g)	M	28 June (5)	3.1	1.5 (2.2)	—
THCY010 (495 mm SVL, 72.2 g)	M	19 September (54)	43.6	11.6 (13.6)	1.065

**Note:**

*Date* is the beginning date (in 2019) and duration (days) an individual was tracked. Distance (*Dist*) is the mean daily distance in meters between relocations divided by days between relocations. Minimum convex polygons (*MCP*) are relative intraseasonal home range sizes (hectares) for individuals with ≥5 relocations. Body sizes are snout-vent length (SVL) in millimeters and mass in grams.

There were apparent advantages and disadvantages of tape telemetry. We observed some instances of sand getting caught in the tape’s leading edge. One snake repeatedly got tangled in dense and exposed vegetation roots at an eroded streambank immediately after tape attachment and another appeared stuck trying to maneuver around a semi-collapsed burrow; we removed the transmitter in both instances. Another snake was found deceased inside a rock crevice with wounds indicative of predation—it is not known if transmitter attachment was related to this event. Conversely, other individuals demonstrated ability to move freely about a heterogeneously complex riparian ecosystem. Through telemetry, we were able to detect individuals using refuges, *e.g*., along steep, rocky slopes with crevices, inside complex high-flow debris piles; (Fig. S4). We seldom detected gartersnakes underneath cover during VES, albeit the many complex or immovable cover objects in the SCRA (Lazaroff, Rosen & Lowe, 2006) limited our efforts to surface scanning. Three adults (two telemetry, one VES) were found by a south-facing canyon wall late season in 2019, potentially indicating nearby hibernacula.

## Discussion

Insight into a common inhabitant of aridland riparian zones like *Thamnophis cyrtopsis* provides useful information. We observed natural and life history traits intertwined with close proximity to surface water, such as foraging and neonatal activity. There was an evident association of temporal consistency in microhabitat assemblages used by *T. cyrtopsis* but with variation by age class and activity level. Concerningly, rapidly changing environmental extremes and disturbances may be affecting population-level dynamics. Taken together, ecological links between behavior, life history, and space use suggests that *T. cyrtopsis* can function as an important model for monitoring how species respond to disturbances and climate change. Such findings may inform how similar aridland riparian fauna may be affected by disturbances and changing systems.

### Environmental relationships and behavioral responses

In lower Sabino Canyon, *T. cyrtopsis* used a complexity of microhabitats that ranged from rocky riparian areas adjacent stream channels, sandy-bottomed pools in bright, open areas, and refuges of exposed tree root systems, rock piles, and woody debris piles deposited from previous high flows. With substantial seasonal shifts in the lower SCRA ecosystem (*i.e*., dry and hot *vs*. humid and wet), it was surprising that microhabitats used by *T. cyrtopsis* did not exhibit temporal variability across seasons or years. Rather, microhabitat composition tended to vary by distance to water—a proxy known to partition activity level and age class among congenerics in similar environments (Halstead et al., 2015; Sprague & Bateman, 2018). Collectively, we provide novel insight into microhabitat assemblages associated with *T. cyrtopsis*.

The link between increased gartersnake activity and environmental covariates, including barometric pressure and relative humidity, likely reflects phenology of life and natural history traits corresponding with seasonal environmental changes during the onset of the summer monsoon (Jones, 1990). Because many pre-monsoon detections occurred at stream microhabitats with standing water—those complex enough to not rapidly dry—and *T. cyrtopsis* were more likely to be active when proximal to water, we interpret this as a period when gartersnakes aggregate and remain close to surface water. The early summer is synchronous with pool contraction and drying, making aquatic prey (*e.g*., fishes) more vulnerable as wetted habitats shrink (Jones & Hensley, 2020). *Thamnophis cyrtopsis* is known to shift behaviors and foraging strategies with seasonal transitions to maximize vulnerabilities of their prey (*e.g*., fish trapped in drying pools, anurans rendered less mobile during metamorphosis; Jones, 1990; Jones & Hensley, 2020). We witnessed such a foraging strategy with how *T. cyrtopsis* captured trapped fish in drying, isolated pools in the SCRA.

Phenology of *T. cyrtopsis* parturition also appears linked with the monsoon season. Breeding usually follows spring emergence (Goldberg, 1998). Though we did not observe parturition or gravid females in this study, we commonly found freshly birthed neonates (*e.g*., based on size and presence of umbilical scar; see Jones & Hensley, 2020) at small, wetted microhabitats with calm or still flows during the period prior to and shortly after the onset of summer rains. Pools that are not too large or dry too quickly likely make good parturition birth sites, especially if they provide prey resources (Holycross & Mitchell, 2020). Similar monsoon-related parturition phenology occurs with other regional snakes (Schuett et al., 2013), including congenerics (Feriche et al., 2016; Ryan et al., 2019; Holycross & Mitchell, 2020).

Precipitation and environmental changes, such as shifts in barometric pressure, during the early monsoon may stimulate fish activity (Stoner, 2004). In turn, this may trigger feeding behaviors in *T. cyrtopsis* until flow is recharged and increased water depths make it more difficult for this generalist species to capture prey (Fleharty, 1967). This could explain why foraging behaviors, by all age classes, were greater when rain occurred within 24 h, but overall water use declined as the active season progressed and aquatic habitats filled (see Fig. 4C). Late-season activity likely reflects ingress towards overwinter refuges; this is supported by post-monsoon observations of multiple individuals at or near rocky, south-facing, upland slopes—often synonymous with snake hibernacula (Holycross & Mitchell, 2020). Autumn mating and overwinter sperm storage is also plausible (Goldberg, 1998). We conclude that *T. cyrtopsis* aggregates at aquatic microhabitats in the hot, early parts summer to feed on vulnerable prey and to give birth, and then remain proximal to such microhabitats until rains intensify and flow resumes, which triggers seasonal dispersal until ingress to upland overwintering sites. Thus, the period preceding intense localized precipitation and recharged stream flow is likely important for *T. cyrtopsis* foraging and reproduction phenology.

### Efficacy of technique

Employing multiple surveying strategies had certain advantages. By emphasizing search-images of behavioral and habitat patterns based on *a priori* understanding of *T. cyrtopsis* natural history (*e.g*., reliance on wetted microhabitats; Jones, 1990; Jones & Hensley, 2020), VES along waterways were productive, especially at isolated slow-flowing pools prior to summer rains. Although *T. cyrtopsis* can venture far from water (Jones & Hensley, 2020), VES detection probability likely declines further from water and into riparian/desertscrub transitional habitat. Using telemetry for slower-moving taxa like snakes helps alleviate search biases of VES and often allows homing to precise locations of individuals (Kingsbury & Robinson, 2016). Telemetry was efficient at acquiring varying behavior and space use data of *T. cyrtopsis* further from water and in shadier locations. Telemetry also provided insight into the use of refuges (*e.g*., complex rockpiles, burrows; see Fig. S4) during periods of inactivity that likely would have been missed during VES. Together, telemetry complemented VES to provide a better understanding of behavior and microhabitat use by *T. cyrtopsis*. We did not track any gravid females in this study, though variation in movement and microhabitat use by gestating congenerics in similar climates is known (Sprague & Bateman, 2018).

We have mixed conclusions about tape as an alternative transmitter attachment methodology in the SCRA or similar xeroriparian systems—largely hindered by drought conditions and habitat complexities. Although external tape attachment may alleviate some complications from internal surgical methodologies (Wylie et al., 2011), tracking duration is limited by ecdysis cycles (Sacerdote-Velat et al., 2014; Sprague & Bateman, 2018). Snakes use refuges during ecdysis as a survival strategy (Chiszar, Smith & Radcliffe, 1993); this adds challenges for retrieving transmitters or determining individual fates. Though, our tracking duration was comparable to similar tape telemetry studies (Wylie et al., 2011; Sacerdote-Velat et al., 2014). Bioclimatic considerations may be most important. Other gartersnake tape telemetry studies occurred in less variable systems with perennial water and more mesic substrates (Wylie et al., 2011; Sprague & Bateman, 2018). Lower Sabino Creek is dominated by xeric, sandy substrates and exposed to seasonal flow cessation and complete drying—these conditions may exacerbate limitations in maneuverability by snakes with external transmitters. Also, some *T. cyrtopsis* exhibited an initial discomfort upon tape attachment, which occurred *in situ* for this study. Mobility hindrances, even in a limited span, may affect survival.

In the SCRA, *T. cyrtopsis* body sizes appeared smaller—specifically thinner—than other Arizona populations (Jones & Hensley, 2020). Our telemetry sample was limited by the lack of adults with sufficient girth, especially after periods of extreme drought, heat, and wildfire. More data could resolve tape telemetry as an attachment alternative or surveillance method complement for assessing spatial ecology in *Thamnophis* or snakes of comparable size or natural history. Because environmental ecosystem (*e.g*., substrate, ephemerality) may be linked with technique performance, researchers should consider habitat complexity and climate in telemetry methodological decisions. Manipulating attachment methods to minimize discomfort or mobility restriction of the individual while ensuring secure transmitter placement (see Sacerdote-Velat et al., 2014) should be further investigated. For example, a flexible yet adequately adhesive tape may provide a fitting compromise between durability and elasticity that is essential for inclement field conditions. For moderate, torpedo-bodied snakes such as *Thamnophis* that swiftly maneuver through tight spaces, we still believe the ventral body placement is best for external attachment. Stockier species with tapered tails (*e.g*., pit vipers) may have added flexibility for dorsal (over bone) attachment in which the chances of transmitter snagging may be reduced. In any case, we recommend that attachment and short-term acclimation monitoring (*e.g*., ≤48 h) occur in a controlled setting to mitigate against any initial discomfort and risks prior to release, when possible.

### Conservation implications

Exploring behavioral and ecological relationships of a species to changing environmental conditions are important to inform effective conservation management. Disturbances and climate change are already cause for concern among sensitive aridland riparian fauna (Jaeger, Olden & Pelland, 2014; Bogan, Boersma & Lytle, 2015; Pilliod et al., 2015; Griffis-Kyle et al., 2018), and evidence suggests some gartersnakes face such challenges (Rossman, Ford & Seigel, 1996; González-Fernández et al., 2018; Holycross & Mitchell, 2020). In the SCRA and surrounding area, wildfire effects, extreme heat and drought, and low winter precipitation recharge rates throughout 2020, as well as unusually high and persistent flooding and flow in 2021, undoubtedly affected resident wildlife populations.

As a representative of semi-arid riparian systems, the dramatic decline in detections of *T. cyrtopsis* immediately after extreme drought and habitat disturbances in the SCRA is concerning. The post-disturbance and temperature linked reduction in observed *T. cyrtopsis* per person visit from 2019 to 2021 were equivalent to a Yavapai Co. (AZ) population decline that proceeded extreme drought and prey extirpation (Fig. 4 in Jones & Hensley, 2020). We witnessed rapid and complete drying of SCRA pools as well as dead or dying fishes within them. Observations of canyon treefrogs (*Hyla arenicolor*) and red-spotted toads (*Anaxyrus punctatus*)—known prey of *T. cyrtopsis* (Jones & Hensley, 2020) and considered common in the SCRA (Lazaroff, Rosen & Lowe, 2006)—were noticeably sparse during lengthy periods of drought and pool drying. Elsewhere, rapid declines in *T. cyrtopsis* populations have been attributed to habitat changes (*e.g*., drought, surface water drying), invasive species, and other climatic effects (Jones & Hensley, 2020). If important microhabitat composition and availability of other resources are altered, or if life history traits become mismatched with phenology or intensity of seasonal changes, then populations can be detrimentally affected (Vitasse et al., 2021). This may be happening concurrently in the SCRA and thus paramount to closely assess how riparian-dependent taxa like *T. cyrtopsis* continue to respond.

Areas with concentrated biodiversity, *e.g*., riparian zones in aridland ecosystems, often yield the greatest number of vulnerable species (Diele-Viegas et al., 2020). Monsoonal climates worldwide are directly linked to primary productivity and biodiversity (Forzieri, Castelli & Vivoni, 2011; Spicer, Farnsworth & Su, 2020), yet changes in climate can bring more extremes (*e.g*., extreme wet or dry conditions) during seasonal monsoons, which may affect adaptation and community structures (Bogan, Boersma & Lytle, 2015; Bridge et al., 2016; Munson et al., 2022). This may be occurring in aridland riparian systems like the SCRA that continue to experience periods of extreme heat, drought, and disturbance effects. Direct and indirect effects of climate change and disturbances on a generalist like *T. cyrtopsis* may act to serve as a canary in the coal mine for how other aridland riparian taxa with similar life history traits might respond. For example, habitat loss and alterations to resource availability are attributed to rapid population declines in threatened Mexican gartersnakes (*T. eques*) and narrow-headed gartersnakes (*T. rufipunctatus*), which have proximal or sympatric overlap with *T. cyrtopsis* in some parts of their distribution (Fleharty, 1967; Ryan et al., 2019; Holycross & Mitchell, 2020). Parturition phenology linked to summer precipitation (*i.e*., seasonal changes in aquatic resources) suggests that these taxa also have some life history commonalities with *T. cyrtopsis* (Rossman, Ford & Seigel, 1996; Blais et al., 2022). Continued disturbances or drastic changes could disrupt phenological patterns or have more direct impacts on fragile populations (Holycross & Mitchell, 2020). Thus, long-term detriment to health or populations of common species like *T. cyrtopsis* are worth monitoring (Lindenmayer et al., 2011), especially if they can model demographic trends and viability in rapidly changing ecosystems during the Anthropocene (Dirzo et al., 2014; Griffis-Kyle, 2016).

## Conclusion

Our findings support that surface water availability and microhabitat heterogeneity play important roles in the life history of riparian-dependent species like *T. cyrtopsis*. Strong spatiotemporal links relative to the extent and timing of available surface water are valuable for foraging and neonatal habitat, and contrary to expectations, microhabitat assemblages used by gartersnakes were consistent across seasons and years. This suggests a reliance upon heterogenous microhabitat structures regardless of developmental age class, and that habitat loss or alteration could be detrimental. Disturbances, including extreme drought, floods, and wildfire, can have rapid effects on populations of riparian-dependent species. Based on dramatic declines in observations from this study and elsewhere (Jones & Hensley, 2020), environmental extremes and disturbances appear to directly or indirectly (*e.g*., impacts to prey base) affect *T. cyrtopsis* populations. Climate change can bring gradual (*e.g*., rising temperature, increase in zero-flow days) or rapid changes (*e.g*., wildfire, flash flooding) that can immediately affect local biodiversity in aridland riparian habitats (Archer & Predick, 2008; Jaeger, Olden & Pelland, 2014; Tecle & Neary, 2015). Populations may face greater extinction risks when climatic extremes or phenological mismatches stress adaptability (Vitasse et al., 2021). Future studies should continue to monitor demographic trends of common, environmentally sensitive species like *T. cyrtopsis* to better understand short- and long-term responses to disturbances and environmental change. Such insight may serve to broadly model challenges that other taxa with similar semi-aquatic life histories might face in changing climates. Employing multiple surveillance techniques, including telemetry, can be advantageous for more comprehensive demographic and response monitoring, but careful methodological consideration is needed to improve application and maximize potential. Informed monitoring across time could result in the development of more effective conservation management strategies in warming and drying ecosystems.

## Supplemental Information

10.7717/peerj.15563/supp-1 Supplemental Information 1 Descriptive statistics of ambient environmental conditions for black-necked gartersnake (*Thamnophis cyrtopsis*) observations in Sabino Canyon Recreation Area, Tucson, Arizona, 2018–2021. Data are partitioned by adults and juveniles (*i.e*., neonates & immature subadults). Ambient variables include *TA* = ambient temperature (°Celsius); *RH* = relative humidity (%); *BP* = barometric pressure (millibars); and *wind* = mean wind speed (meters/second).

10.7717/peerj.15563/supp-2 Supplemental Information 2 Summary of black-necked gartersnake (*Thamnophis cyrtopsis*) behaviors observed in Sabino Canyon Recreation Area, Tucson, Arizona, 2018–2021. Data are partitioned by developmental age classes, adults and juveniles (*i.e*., neonates & immature subadults) and sorted by possible opportunities (*n*), times observed (obs), and percentage (%) observed per opportunity per age class. Individuals could exhibit >1 behavior per observation. Significance of association between age class and behavior type is noted by results of Fisher’s Exact Tests (*p*).

10.7717/peerj.15563/supp-3 Supplemental Information 3 Effects on behaviors by black-necked gartersnakes (*Thamnophis cyrtopsis*) in Sabino Canyon Recreation Area, Tucson, Arizona, 2018–2021. Selection of logistic generalized linear models on behaviors: (A) alertness; (B) foraging; (C) inactivity; (D) movement; and (E) water use. *Model* represents the model covariates; *k* is the number of model parameters; *AICc* is the corrected Akaike Information Criterion score; *∆AICc* is the change in AICc scores; *loglik* is the model maximum log likelihood; and *R^2^* is McFadden’s pseudo r-squared value. Behaviors and covariates are described in Dataset S1.

10.7717/peerj.15563/supp-4 Supplemental Information 4 Principal Components Analysis (PCA) of quantitative microhabitat composition characteristics used by black-necked gartersnakes (*Thamnophis cyrtopsis*) in Sabino Canyon Recreation Area, Tucson, Arizona, 2018–2021. Principal components with eigenvalues >1 and explained variance ≥10% are considered important.

10.7717/peerj.15563/supp-5 Supplemental Information 5 Composition of microhabitat assemblages used by black-necked gartersnakes (*Thamnophis cyrtopsis*) in Sabino Canyon Recreation Area, Tucson, Arizona, 2018–2021. Selection of logistic generalized linear models for (A) activity level (*i.e*., active *vs*. inactive); (B) developmental age class (adults *vs*. juveniles); and (C) detection method (telemetry *vs* visual encounter). *Model* represents the model covariates; *k* is the number of model parameters; *AICc* is corrected Akaike Information Criterion score; *∆AICc* is the change in AICc scores; *loglik* is the model log likelihood; and *R^2^* is McFadden’s pseudo r-squared value. Microhabitat plot parameters are described in Dataset S1.

10.7717/peerj.15563/supp-6 Supplemental Information 6 Examples of stream microhabitats in lower Sabino Canyon Recreation Area, Tucson, Arizona. (**A**) An eroded bank pool in early July 2019 prior to monsoon flow recharge with a neonate black-necked gartersnake (*Thamnophis cyrtopsis*) positioned on an emerged rock (yellow circle); and (**C**) the same locality in late September 2020 after flooding deposited slurry from the Bighorn Wildfire; (**B**) continuous flow in riffle microhabitat during 2021 monsoonal recharge; and (**D**) isolated drying pool occupied by several neonate *T. cyrtopsis* prior to monsoonal recharge in early July 2019. Photo credits: B. Blais.

10.7717/peerj.15563/supp-7 Supplemental Information 7 Operational environmental conditions for surface-active black-necked gartersnakes (*Thamnophis cyrtopsis*) in Sabino Canyon Recreation Area, Tucson, Arizona, 2018–2021. Data are partitioned by (A) relative humidity (±0.1%); and (B) ambient temperature (±0.1 °C). Vertical lines indicate median values. Blue colors represent surface active status (*e.g*., moving, water use) and red colors indicates inactive status (*e.g*., basking, refuge use).

10.7717/peerj.15563/supp-8 Supplemental Information 8 Relationship between mean daily distance travelled (in meters) and telemetry tracking duration (days) of black-necked gartersnakes (*Thamnophis cyrtopsis*) in Sabino Canyon Recreation Area, Tucson, Arizona, 2019. Linear regression line with standard error (shading); *p* = 0.122; adjusted *R^2^* = 0.363.

10.7717/peerj.15563/supp-9 Supplemental Information 9 Large debris pile refuge in Sabino Creek floodplain used by an adult male black-necked gartersnake (*Thamnophis cyrtopsis*) found *via* external “tape” radio telemetry in September 2019. Photo credit: B. Blais.

10.7717/peerj.15563/supp-10 Supplemental Information 10 Datasets used for analyses of survey-level, spatial, behavioral, microhabitat use data by black-necked gartersnakes (*Thamnophis cyrtopsis*) in Sabino Canyon Recreation Area, Tucson, Arizona, 2018–2021. Datasets are sorted by sheets. A key of variables and descriptions is included.

## Additional Information and Declarations

### Field Study Permissions

The following information was supplied relating to field study approvals (*i.e*., approving body and any reference numbers): Fieldwork was conducted under scientific research permits from Arizona Game and Fish Department and USDA Forest Service (SUP1841).

### Data Availability

The following information was supplied regarding data availability: Raw data is available in the Supplemental Files. Dataset S1 provides the datasets used for analyses in this project. A key of variables and descriptions is also included.
