# Supplementary material for: Effects of disturbances and environmental changes on an aridland riparian generalist
Source: PeerJ. 2023 Jun 19;11:e15563. doi: 10.7717/peerj.15563 (PMC10286802; doi:10.7717/peerj.15563)
Supplement: Supplemental Information 1 — Data are partitioned by adults and juveniles (i.e., neonates & immature subadults). Ambient variables include TA = ambient temperature (°Celsius); RH = relative humidity (%); BP = barometric pressure (millibars); and wind = mean wind speed (meters/second). [file peerj-11-15563-s001.docx]

**Supplemental Table S1. Descriptive statistics of ambient environmental conditions for black-necked gartersnake (*Thamnophis cyrtopsis*) observations in Sabino Canyon Recreation Area, Tucson, Arizona, 2018–2021.** Data are partitioned by adults and juveniles (i.e., neonates & immature subadults). Ambient variables include *TA* = ambient temperature (°Celsius); *RH* = relative humidity (%); *BP* = barometric pressure (millibars); and *wind* = mean wind speed (meters/second).

| **Age class** | **Ambient** | **Min** | **Max** | **Median** | **Mean (±SD)** |
| --- | --- | --- | --- | --- | --- |
| Adults (*n* = 45) | TA (°C) | 24.7 | 37.8 | 33.2 | 32.9 (±4.0) |
|  | RH (%) | 15.6 | 58.4 | 25.7 | 29.9 (±10.9) |
|  | BP (mb) | 914.1 | 926.3 | 920.6 | 920.6 (±3.0) |
|  | wind (m/s) | 0.0 | 1.7 | 0.0 | 0.3 (±0.4) |
| Juveniles (*n* = 47) | TA (°C) | 20.7 | 37.1 | 31.9 | 31.4 (±3.5) |
|  | RH (%) | 22.0 | 54.0 | 42.7 | 39.9 (±9.6) |
|  | BP (mb) | 917.7 | 925.2 | 921.6 | 921.4 (±2.2) |
|  | wind (m/s) | 0.0 | 0.8 | 0.2 | 0.2 (±0.2) |
