# Supplementary material for: Effects of disturbances and environmental changes on an aridland riparian generalist
Source: PeerJ. 2023 Jun 19;11:e15563. doi: 10.7717/peerj.15563 (PMC10286802; doi:10.7717/peerj.15563)
Supplement: Supplemental Information 2 — Data are partitioned by developmental age classes, adults and juveniles (i.e., neonates & immature subadults) and sorted by possible opportunities (n), times observed (obs), and percentage (%) observed per opportunity per age class. Individuals could exhibit >1 behavior per observation. Significance of association between age class and behavior type is noted by results of Fisher’s Exact Tests (p). [file peerj-11-15563-s002.docx]

Table S2. Summary of black-necked gartersnake (*Thamnophis cyrtopsis*) behaviors observed in Sabino Canyon Recreation Area, Tucson, Arizona, 2018–2021. Data are partitioned by developmental age classes, adults and juveniles (i.e., neonates & immature subadults) and sorted by possible opportunities (*n*), times observed (obs), and percentage (%) observed per opportunity per age class. Individuals could exhibit >1 behavior per observation. Significance of association between age class and behavior type is noted by results of Fisher’s Exact Tests (*p*).

|  |  |  | | *Age class* | | | | |
| --- | --- | --- | --- | --- | --- | --- | --- | --- |
| Behaviors | Adult | | | | Juveniles | | |  |
|  | *n* | | obs | % | *n* | obs | % | *p* |
| Alert (*n* = 82) | 42 | | 18 | 42.9 | 40 | 16 | 40.0 | 0.826 |
| Foraging (*n* = 82) | 42 | | 10 | 23.8 | 40 | 20 | 50.0 | 0.021 |
| Inactive (*n* = 89) | 43 | | 16 | 37.2 | 46 | 2 | 4.4 | <0.001 |
| Moving (*n* = 88) | 43 | | 14 | 32.6 | 45 | 16 | 35.6 | 0.824 |
| Water use (*n* = 87) | 43 | | 15 | 34.9 | 44 | 31 | 70.5 | 0.001 |
