# Supplementary material for: Effects of disturbances and environmental changes on an aridland riparian generalist
Source: PeerJ. 2023 Jun 19;11:e15563. doi: 10.7717/peerj.15563 (PMC10286802; doi:10.7717/peerj.15563)
Supplement: Supplemental Information 3 — Selection of logistic generalized linear models on behaviors: (A) alertness; (B) foraging; (C) inactivity; (D) movement; and (E) water use. Model represents the model covariates; k is the number of model parameters; AICc is the corrected Akaike Information Criterion score; ∆AICc is the change in AICc scores; loglik is the model maximum log likelihood; and R2 is McFadden’s pseudo r-squared value. Behaviors and covariates are described in Dataset S1. [file peerj-11-15563-s003.docx]

**Table S3. Effects on behaviors by black-necked gartersnakes (*Thamnophis cyrtopsis*) in Sabino Canyon Recreation Area, Tucson, Arizona, 2018–2021.** Selection of logistic generalized linear models on behaviors: A) alertness; B) foraging; C) inactivity; D) movement; and E) water use. *Model* represents the model covariates; *k* is the number of model parameters; *AICc* is the corrected Akaike Information Criterion score; *∆AICc* is the change in AICc scores; *loglik* is the model maximum log likelihood; and *R^2^* is McFadden’s pseudo r-squared value. Behaviors and covariates are described in Supplemental Dataset S1.

| **Behavior** | **Model** | **k** | **AICc** | **∆AICc** | **loglik** | **R^2^** |
| --- | --- | --- | --- | --- | --- | --- |
| **A)** Alert | ~ season +RH +W +rain | 6 | 45.01 | 0.00 | -15.48 | 0.53 |
|  | ~ season +W +rain | 5 | 47.99 | 2.98 | -18.30 | 0.45 |
|  | ~ season +dH2O +TA +RH +BP +W +rain | 11 | 59.41 | 14.40 | -15.04 | 0.54 |
|  | ~ W +rain | 3 | 67.49 | 22.48 | -30.48 | 0.08 |
|  | ~ 1 | 1 | 113.32 | 68.32 | -55.64 | -- |
| **B)** Foraging | ~ age +season +dH2O +RH +rain | 9 | 51.70 | 0.00 | -14.71 | 0.50 |
|  | ~ season +dH2O +RH | 7 | 52.95 | 1.24 | -18.20 | 0.38 |
|  | ~ age +season +dH2O +TA +RH +BP +rain | 11 | 53.05 | 1.34 | -12.22 | 0.58 |
|  | ~ age +dH2O +RH | 6 | 55.29 | 3.58 | -20.71 | 0.29 |
|  | ~ 1 | 1 | 109.75 | 58.05 | -53.85 | -- |
| **C)** Inactive | ~ TA +BP | 3 | 49.50 | 0.00 | -21.50 | 0.16 |
|  | ~ age +TA +RH +BP +W | 6 | 52.37 | 2.87 | -19.23 | 0.24 |
|  | ~ age +season +TA +RH +BP +W +rain | 9 | 54.21 | 4.71 | -15.91 | 0.37 |
|  | ~ age +season +dH2O +TA +RH +BP +W +rain | 12 | 56.21 | 6.71 | -12.00 | 0.53 |
|  | ~ 1 | 1 | 91.67 | 42.17 | -44.81 | -- |
| **D)** Moving | ~ dH2O +RH | 5 | 62.79 | 0.00 | -25.74 | 0.23 |
|  | ~ dH2O +BP | 5 | 64.38 | 1.59 | -26.55 | 0.23 |
|  | ~ dH2O +RH +BP | 6 | 65.10 | 2.31 | -25.61 | 0.24 |
|  | ~ season +dH2O +TA +RH +BP +W | 10 | 73.45 | 10.66 | -23.97 | 0.28 |
|  | ~ 1 | 1 | 114.97 | 52.19 | -56.46 | -- |
| **E)** Water use | ~ season +TA | 4 | 68.63 | 0.00 | -29.89 | 0.15 |
|  | ~ age +season +TA | 5 | 70.15 | 1.52 | -29.42 | 0.16 |
|  | ~ age +season +TA +RH +BP +W +rain | 9 | 72.68 | 4.05 | -25.09 | 0.25 |
|  | ~ age +season | 4 | 104.69 | 36.07 | -48.10 | 0.20 |
|  | ~1 | 1 | 122.37 | 53.74 | -60.16 | -- |

*Model parameter abbreviations*: *age* = age class (adult, juvenile); *BP* = ambient barometric pressure (millibars); *dH2O* = distance to water (0, <1, 1–5, >5 m); *RH* = ambient relative humidity (%); *rain* = rain recency (none; ≤24h); *season* = season (pre-monsoon, monsoon, post-monsoon); *TA* = ambient temperature (°C); *W* = ambient wind (m/s).
