# Supplementary material for: Effects of disturbances and environmental changes on an aridland riparian generalist
Source: PeerJ. 2023 Jun 19;11:e15563. doi: 10.7717/peerj.15563 (PMC10286802; doi:10.7717/peerj.15563)
Supplement: Supplemental Information 4 — Principal components with eigenvalues >1 and explained variance ≥10% are considered important. [file peerj-11-15563-s004.docx]

**Table S4. Principal Components Analysis (PCA) of quantitative microhabitat composition characteristics used by black-necked gartersnakes (*Thamnophis cyrtopsis*) in Sabino Canyon Recreation Area, Tucson, Arizona, 2018–2021.** Principal components with eigenvalues >1 and explained variance ≥10% are considered important.

| **Principal Components** | **Eigenvalue** | **Variance (%)** | **Cumulative variance (%)** |
| --- | --- | --- | --- |
| PC1 | 3.763 | 26.881 | 26.881 |
| PC2 | 2.238 | 15.989 | 42.870 |
| PC3 | 2.004 | 14.312 | 57.181 |
| PC4 | 1.550 | 11.069 | 68.250 |
| PC5 | 1.218 | 8.700 | 76.950 |
| PC6 | 1.043 | 7.448 | 84.398 |
| PC7 | 0.715 | 5.106 | 89.504 |
| PC8 | 0.452 | 3.231 | 92.736 |
| PC9 | 0.380 | 2.715 | 95.451 |
| PC10 | 0.196 | 1.397 | 96.848 |
| PC11 | 0.178 | 1.271 | 98.119 |
| PC12 | 0.140 | 1.000 | 99.119 |
| PC13 | 0.083 | 0.590 | 99.708 |
| PC14 | 0.041 | 0.292 | 100.000 |
