# Supplementary material for: Effects of disturbances and environmental changes on an aridland riparian generalist
Source: PeerJ. 2023 Jun 19;11:e15563. doi: 10.7717/peerj.15563 (PMC10286802; doi:10.7717/peerj.15563)
Supplement: Supplemental Information 5 — Selection of logistic generalized linear models for (A) activity level (i.e., active vs. inactive); (B) developmental age class (adults vs. juveniles); and (C) detection method (telemetry vs visual encounter). Model represents the model covariates; k is the number of model parameters; AICc is corrected Akaike Information Criterion score; ∆AICc is the change in AICc scores; loglik is the model log likelihood; and R2 is McFadden’s pseudo r-squared value. Microhabitat plot parameters are described in Dataset S1. [file peerj-11-15563-s005.docx]

**Table S5**. **Composition of microhabitat assemblages used by black-necked gartersnakes (*Thamnophis cyrtopsis*) in Sabino Canyon Recreation Area, Tucson, Arizona, 2018–2021**. Selection of logistic generalized linear models for A) activity level (i.e., active vs. inactive); B) developmental age class (adults vs. juveniles); and C) detection method (telemetry vs visual encounter). *Model* represents the model covariates; *k* is the number of model parameters; *AICc* is corrected Akaike Information Criterion score; *∆AICc* is the change in AICc scores; *loglik* is the model log likelihood; and *R^2^* is McFadden’s pseudo r-squared value. Microhabitat plot parameters are described in Supplemental Dataset S1.

| **Behavior** | **Model** | **k** | **AICc** | **∆AICc** | **loglik** | **R^2^** |
| --- | --- | --- | --- | --- | --- | --- |
| **A)** Active | ~ shade +H2O +rock +woody +trees +can | 7 | 39.33 | 0.00 | -11.61 | 0.66 |
|  | ~ shade +H2O +rock +woody +trees +can +cover | 9 | 43.37 | 4.04 | -10.92 | 0.68 |
|  | ~ shade +H2O +woody | 4 | 45.65 | 6.33 | -18.50 | 0.54 |
|  | ~ shade +H2O +rock +woody +trees +can +season +cover | 10 | 46.06 | 6.73 | -10.83 | 0.68 |
|  | ~ 1 | 1 | 103.26 | 63.93 | -50.61 | -- |
| **B)** Age class | ~ shade +H2O +rock +woody +trees +can | 7 | 72.11 | 0.00 | -28.02 | 0.35 |
|  | ~ shade +H2O +rock +woody +trees +can +season +cover | 11 | 74.32 | 2.96 | -23.52 | 0.45 |
|  | ~ H2O +woody +can | 4 | 75.17 | 3.80 | -33.31 | 0.38 |
|  | ~ H2O +can | 3 | 77.08 | 5.72 | -35.38 | 0.34 |
|  | ~ 1 | 1 | 144.82 | 73.45 | -71.39 | -- |
| **C)** Method | ~ shade +H2O | 3 | 52.76 | 0.00 | -23.20 | 0.43 |
|  | ~ shade +H2O +woody +can | 5 | 53.27 | 0.51 | -21.09 | 0.44 |
|  | ~ shade +H2O +woody | 4 | 54.45 | 1.69 | -22.91 | 0.44 |
|  | ~ shade +H2O +rock +woody +trees +can | 7 | 58.08 | 5.32 | -20.98 | 0.45 |
|  | ~ 1 | 1 | 114.81 | 62.06 | -56.39 | -- |

*Model parameter abbreviations*: *can* = mean overhead canopy cover (%); *cover* = height class of plot cover vegetation (none, <1m, 1–2m, >2m); *rock* = rock %; *season* = season (dry, wet); *shade* = shade %; *trees* = trees/roots %; *woody* = woody debris %; *H2O* = water %.
