# Supplementary figures and images for: Effects of disturbances and environmental changes on an aridland riparian generalist
Source: PeerJ. 2023 Jun 19;11:e15563. doi: 10.7717/peerj.15563 (PMC10286802; doi:10.7717/peerj.15563)

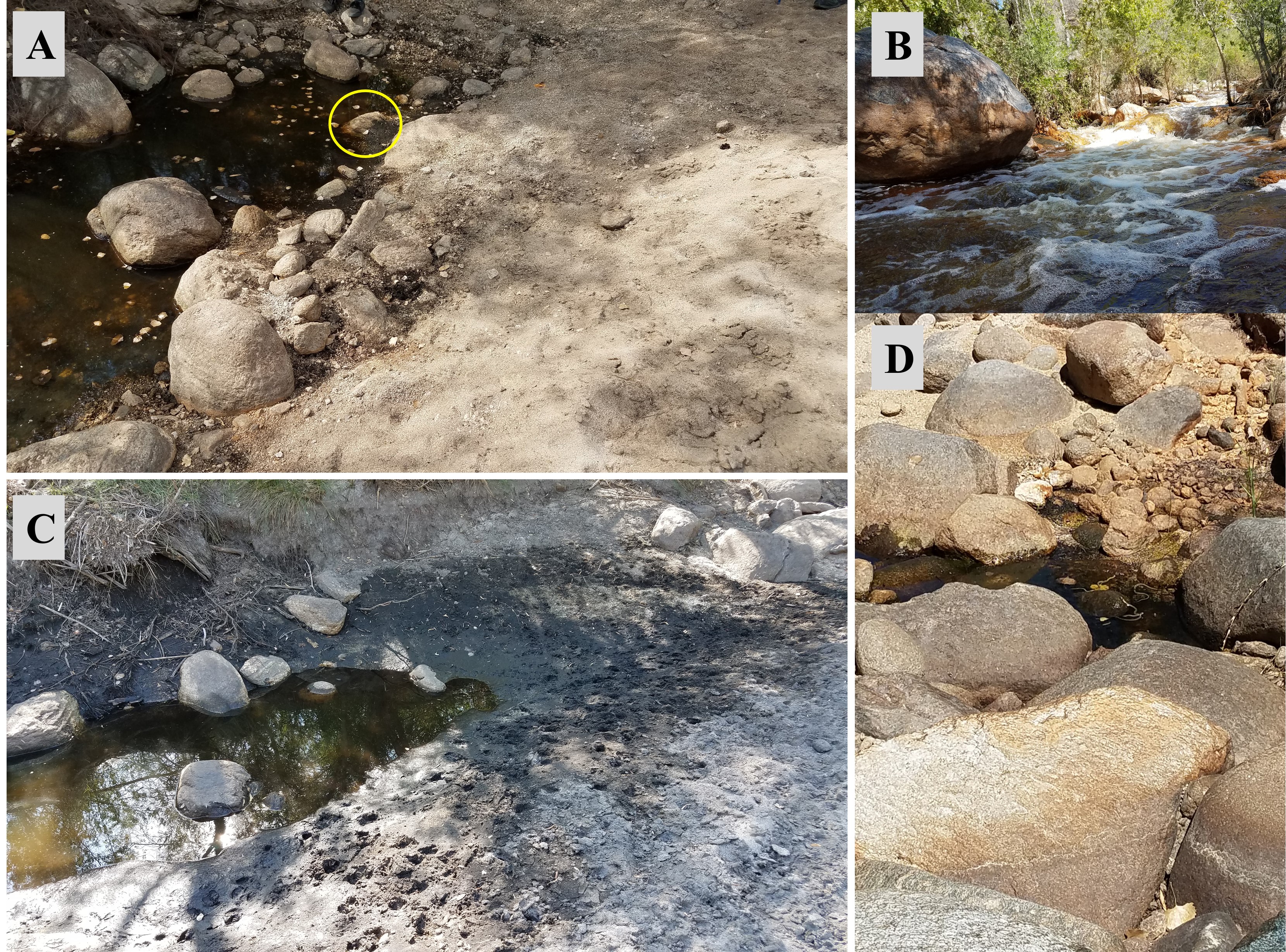

Supplement: Supplemental Information 6 — (A) An eroded bank pool in early July 2019 prior to monsoon flow recharge with a neonate black-necked gartersnake (Thamnophis cyrtopsis) positioned on an emerged rock (yellow circle); and (C) the same locality in late September 2020 after flooding deposited slurry from the Bighorn Wildfire; (B) continuous flow in riffle microhabitat during 2021 monsoonal recharge; and (D) isolated drying pool occupied by several neonate T. cyrtopsis prior to monsoonal recharge in early July 2019. Photo credits: B. Blais. [file peerj-11-15563-s006.jpg]

**A**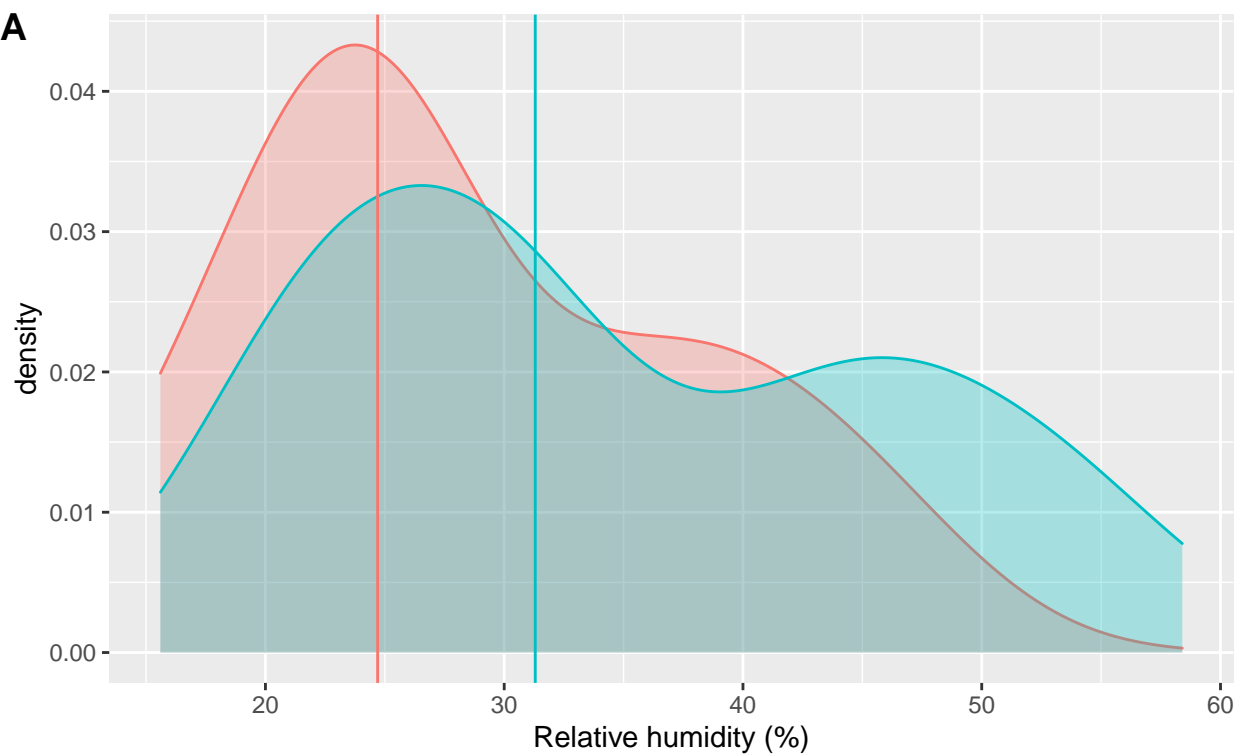**B**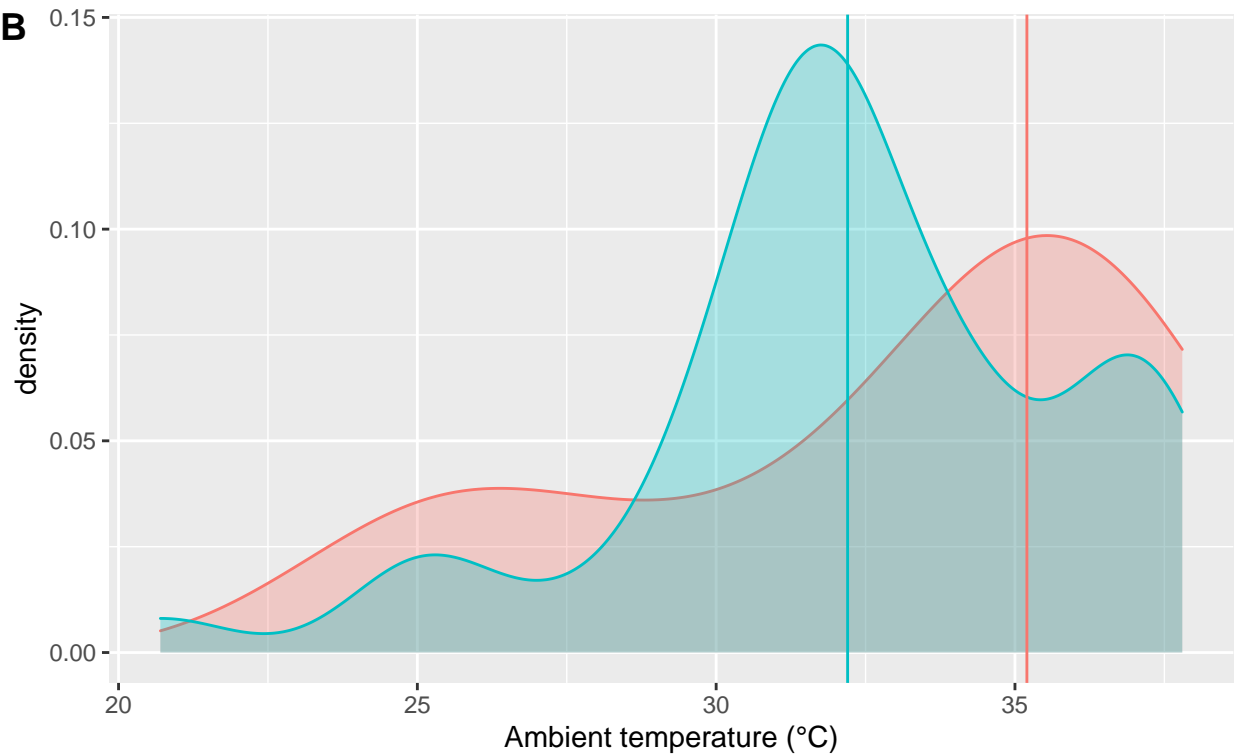

Supplement: Supplemental Information 7 — Data are partitioned by (A) relative humidity (±0.1%); and (B) ambient temperature (±0.1 °C). Vertical lines indicate median values. Blue colors represent surface active status (e.g., moving, water use) and red colors indicates inactive status (e.g., basking, refuge use). [file peerj-11-15563-s007.pdf]

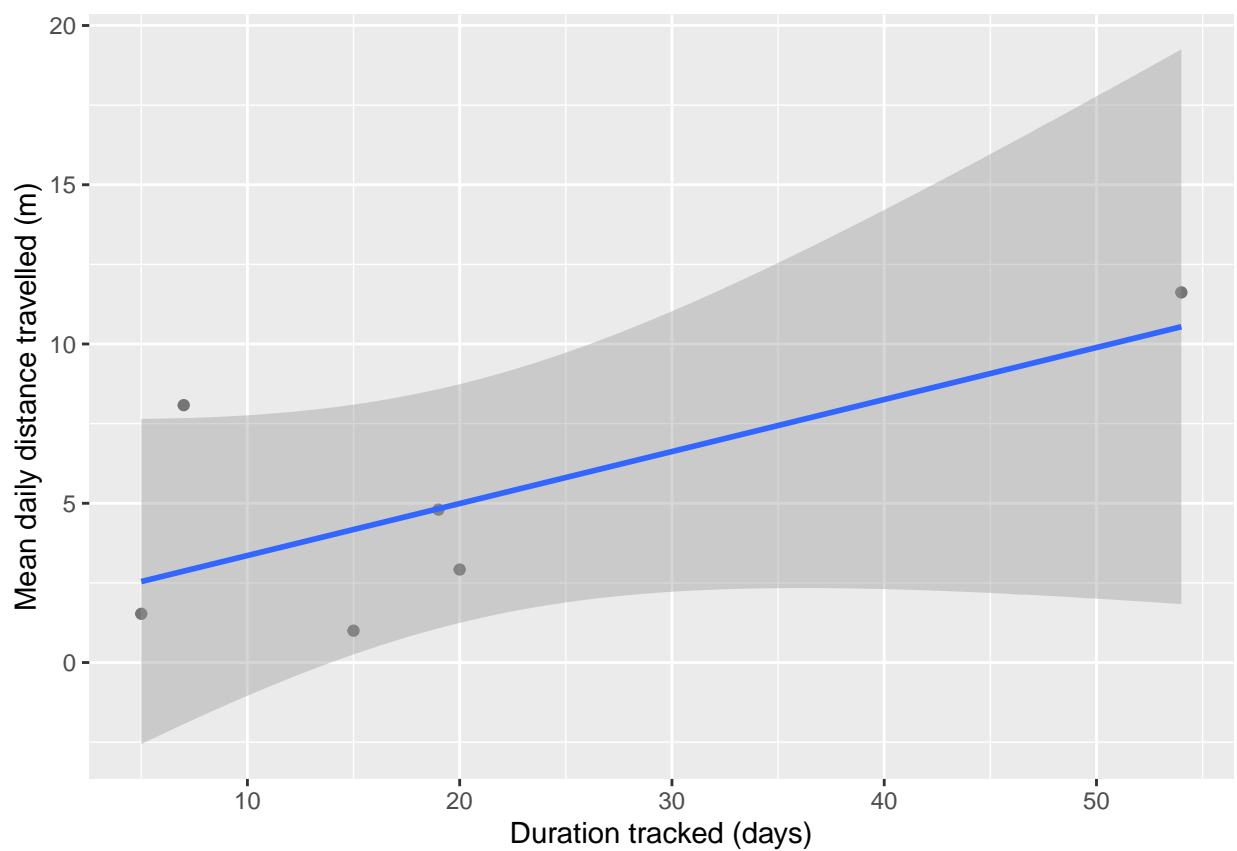

Supplement: Supplemental Information 8 — Linear regression line with standard error (shading); p = 0.122; adjusted R2 = 0.363. [file peerj-11-15563-s008.pdf]

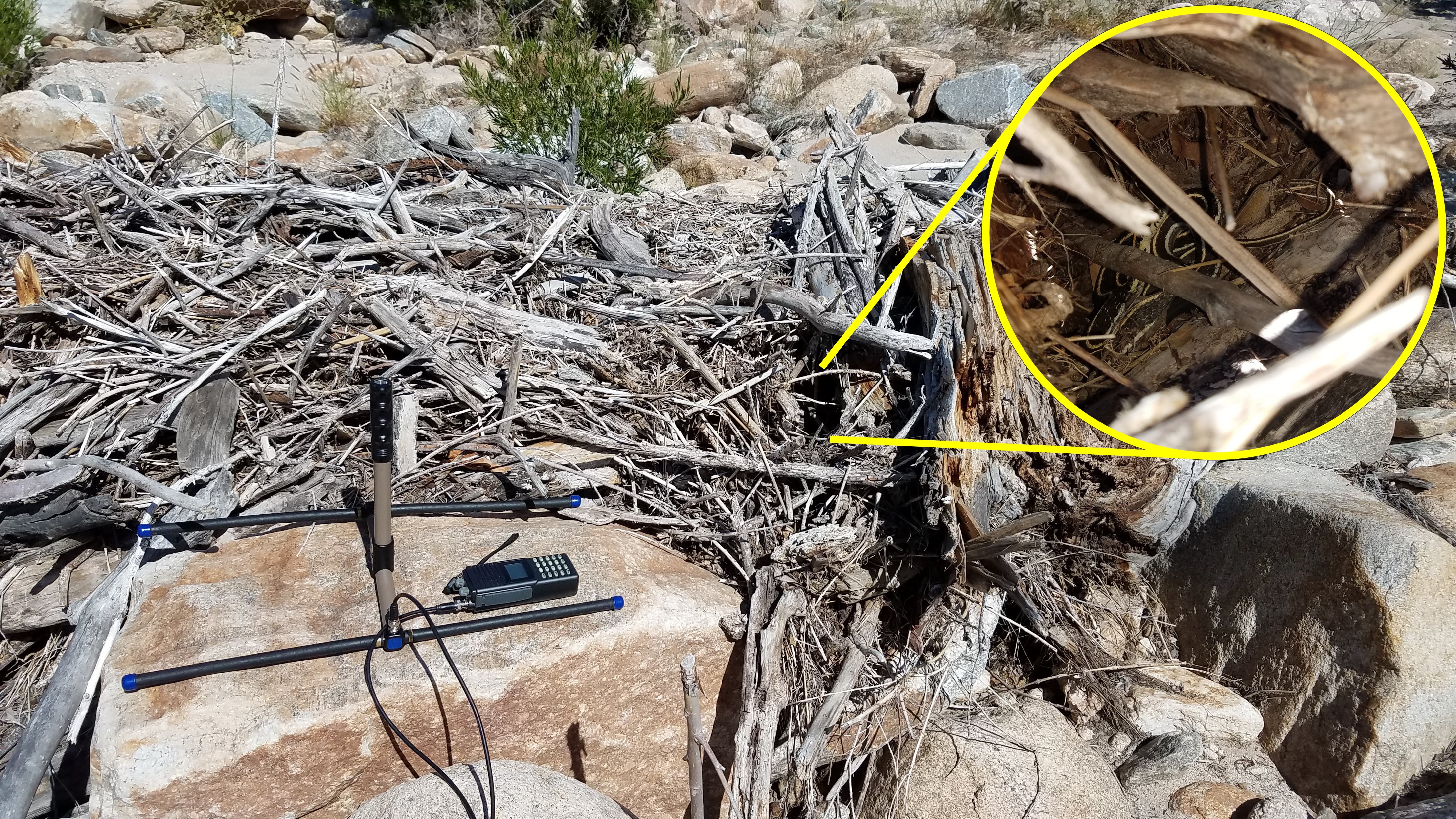

Supplement: Supplemental Information 9 — Photo credit: B. Blais. [file peerj-11-15563-s009.jpg]
